# Retraction: Virtual reality-guided aortic valve leaflet reconstruction for type 0 bicuspid aortic stenosis

**DOI:** 10.1093/icvts/ivac281

**Published:** 2022-12-02

This is a retraction of: Takanori Tsujimoto, Takeo Tedoriya, Kenji Okada, Virtual reality-guided aortic valve leaflet reconstruction for type 0 bicuspid aortic stenosis, *Interactive CardioVascular and Thoracic Surgery*, Volume 34, Issue 6, June 2022, Pages 1152–1154, https://doi.org/10.1093/icvts/ivab353

After a reader raised concerns about the ethical oversight of this case report in September 2022, both Kobe University and Ageo Central General Hospital investigated. The university ultimately noted two issues: patient consent had not been obtained prior to article publication and Institutional Review Board approval had not been received for the use of the Vesalius3D software, despite the software not being approved for medical use by the pharmaceutical authority of the Japanese government. Based on this information, the hospital recommended retracting this case report, and the authors and editors agree. The authors apologize to the readers of *Interactive CardioVascular and Thoracic Surgery* for these oversights.

